# Supplementary figures and images for: Pseudotargeted lipidomics analysis of scoparone on glycerophospholipid metabolism in non-alcoholic steatohepatitis mice by LC-MRM-MS
Source: PeerJ. 2024 May 21;12:e17380. doi: 10.7717/peerj.17380 (PMC11122033; doi:10.7717/peerj.17380)

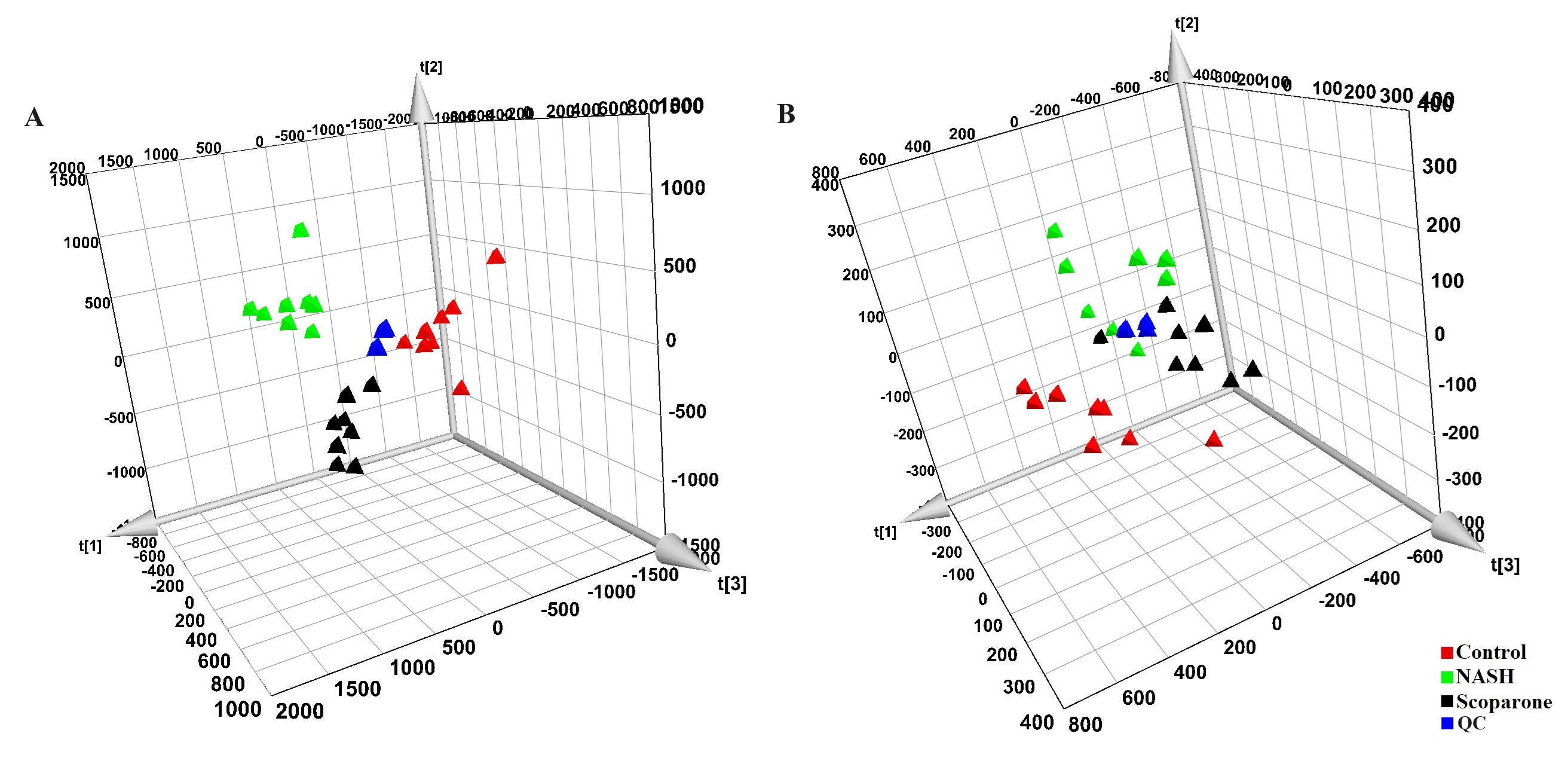

Supplement: Figure S1 [file peerj-12-17380-s001.png]

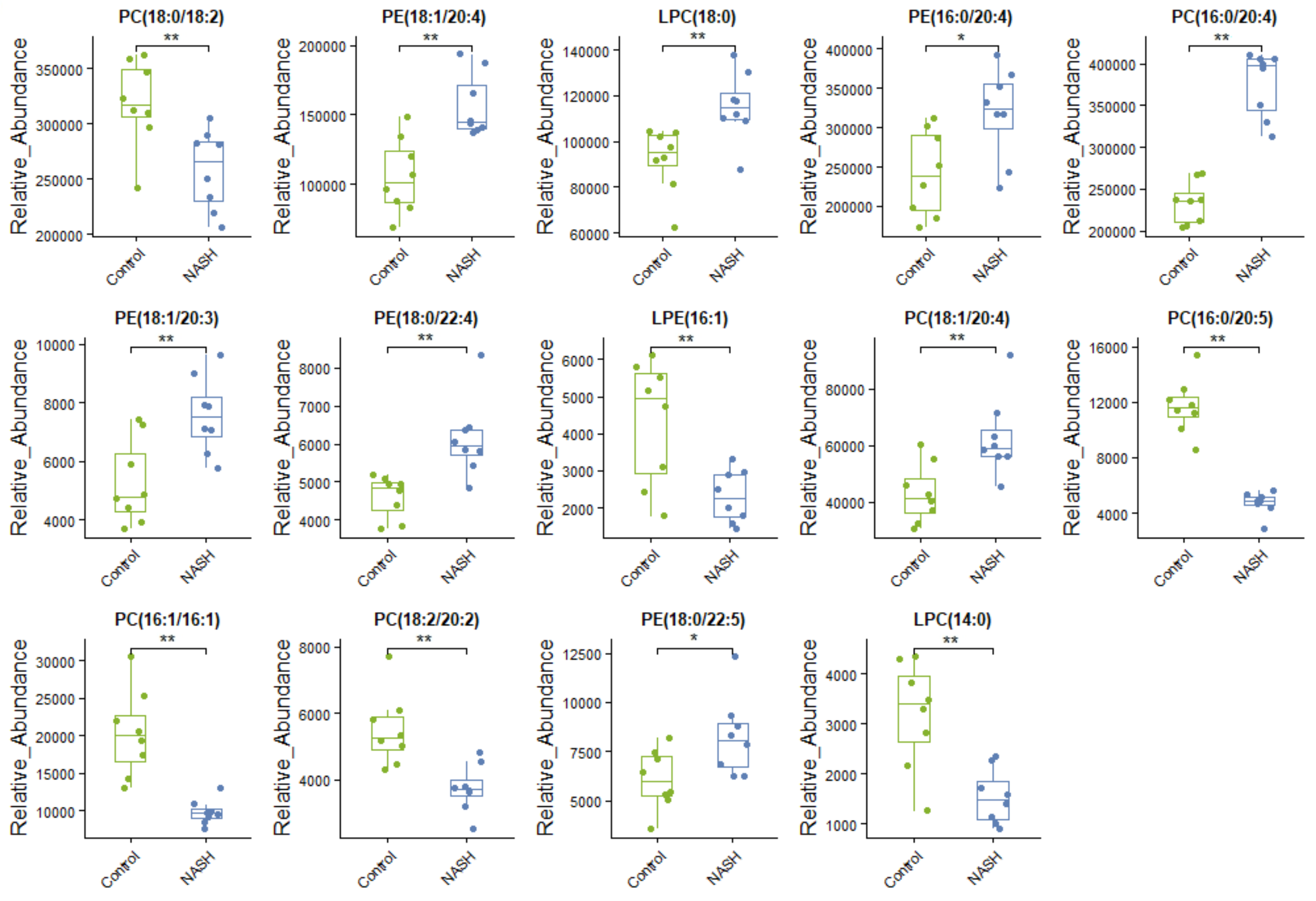

Supplement: Figure S2 — *P < 0.05 and **P < 0.01 compared with the control group. [file peerj-12-17380-s002.png]
