# Supplementary material for: Pseudotargeted lipidomics analysis of scoparone on glycerophospholipid metabolism in non-alcoholic steatohepatitis mice by LC-MRM-MS
Source: PeerJ. 2024 May 21;12:e17380. doi: 10.7717/peerj.17380 (PMC11122033; doi:10.7717/peerj.17380)
Supplement: Table S1 [file peerj-12-17380-s003.pdf]

Table S1 The internal standard substance information

Name	Molecular formula	Molecular weight	Manufacturer
17:0-14:1 PC-d5	C39H71D5NO8P	722.5622	Avanti
17:0-16:1 PC-d5	C41H75D5NO8P	750.5935	Avanti
17:0-18:1 PC-d5	C43H79D5NO8P	778.6248	Avanti
17:0-20:3 PC-d5	C45H79D5NO8P	802.6248	Avanti
17:0-22:4 PC-d5	C47H81D5NO8P	828.6405	Avanti
17:0-14:1 PE-d5	C36H65D5NO8P	680.5153	Avanti
17:0-16:1 PE-d5	C38H69D5NO8P	708.5466	Avanti
17:0-18:1 PE-d5	C40H73D5NO8P	736.5779	Avanti
17:0-20:3 PE-d5	C42H73D5NO8P	760.5779	Avanti
17:0-22:4 PE-d5	C44H75D5NO8P	786.5935	Avanti
17:0-14:1 PG-d5 (Na Salt)	C37H65D5NaO10P	733.4918	Avanti
17:0-16:1 PG-d5 (Na Salt)	C39H69D5NaO10P	761.5231	Avanti
17:0-18:1 PG-d5 (Na Salt)	C41H73D5NaO10P	789.5544	Avanti
17:0-20:3 PG-d5 (Na Salt)	C43H73D5NaO10P	813.5544	Avanti
17:0-22:4 PG-d5 (Na Salt)	C45H75D5NaO10P	839.5701	Avanti
17:0-14:1 PS-d5 (Na Salt)	C37H64D5NNaO10P	746.4871	Avanti
17:0-16:1 PS-d5 (Na Salt)	C39H68D5NNaO10P	774.5184	Avanti
17:0-18:1 PS-d5 (Na Salt)	C41H72D5NNaO10P	802.5497	Avanti
17:0-20:3 PS-d5 (Na Salt)	C43H72D5NNaO10P	826.5497	Avanti
17:0-22:4 PS-d5 (Na Salt)	C45H74D5NNaO10P	852.5653	Avanti
17:0-14:1 PI-d5 (NH4 Salt)	C40H73D5NO13P	816.5525	Avanti
17:0-16:1 PI-d5 (NH4 Salt)	C42H77D5NO13P	844.5838	Avanti
17:0-18:1 PI-d5 (NH4 Salt)	C44H81D5NO13P	872.6151	Avanti
17:0-20:3 PI-d5 (NH4 Salt)	C46H81D5NO13P	896.6151	Avanti
17:0-22:4 PI-d5 (NH4 Salt)	C48H83D5NO13P	922.6307	Avanti
15:0 Lyso PI-d5 (NH4 Salt)	C24H45D5NO12P	580.3384	Avanti
17:0 Lyso PI-d5 (NH4 Salt)	C26H49D5NO12P	608.3697	Avanti
19:0 Lyso PI-d5 (NH4 Salt)	C28H53D5NO12P	636.401	Avanti
15:0 Lyso PS-d5 (Na Salt)	C21H36D5NNaO9P	510.273	Avanti
17:0 Lyso PS-d5 (Na Salt)	C23H40D5NNaO9P	538.3043	Avanti
19:0 Lyso PS-d5 (Na Salt)	C25H44D5NNaO9P	566.3356	Avanti
15:0 Lyso PG-d5 (Na Salt)	C21H37D5NaO9P	497.2778	Avanti
17:0 Lyso PG-d5 (Na Salt)	C23H41D5NaO9P	525.3091	Avanti
19:0 Lyso PG-d5 (Na Salt)	C25H45D5NaO9P	553.3404	Avanti
15:0 Lyso PC-d5	C23H43D5NO7P	486.3482	Avanti
17:0 Lyso PC-d5	C25H47D5NO7P	514.3795	Avanti
19:0 Lyso PC-d5	C27H51D5NO7P	542.4108	Avanti
15:0 Lyso PE-d5	C20H37D5NO7P	444.3013	Avanti
17:0 Lyso PE-d5	C22H41D5NO7P	472.3326	Avanti
19:0 Lyso PE-d5	C24H45D5NO7P	500.3639	Avanti
LPA 17:0 (Na Salt)	C20H40O7PNa	446.49	Avanti
PA(17:0/17:0)	C37H72O8PNa	698.93	Avanti
